# A Benchmark Dataset for RSVP-Based Brain–Computer Interfaces

**DOI:** 10.3389/fnins.2020.568000

**Published:** 2020-10-02

**Authors:** Shangen Zhang, Yijun Wang, Lijian Zhang, Xiaorong Gao

**Affiliations:** ^1^School of Computer and Communication Engineering, University of Science and Technology Beijing, Beijing, China; ^2^Beijing Key Laboratory of Knowledge Engineering for Materials Science, Beijing, China; ^3^State Key Laboratory on Integrated Optoelectronics, Institute of Semiconductors, Chinese Academy of Sciences, Beijing, China; ^4^Beijing Machine and Equipment Institute, Beijing, China; ^5^Department of Biomedical Engineering, School of Medicine, Tsinghua University, Beijing, China

**Keywords:** rapid serial visual presentation, brain–computer interface, electroencephalogram, target detection, public dataset, event-related potential

## Abstract

This paper reports on a benchmark dataset acquired with a brain–computer interface (BCI) system based on the rapid serial visual presentation (RSVP) paradigm. The dataset consists of 64-channel electroencephalogram (EEG) data from 64 healthy subjects (sub1,…, sub64) while they performed a target image detection task. For each subject, the data contained two groups (“A” and “B”). Each group contained two blocks, and each block included 40 trials that corresponded to 40 stimulus sequences. Each sequence contained 100 images presented at 10 Hz (10 images per second). The stimulus images were street-view images of two categories: target images with human and non-target images without human. Target images were presented randomly in the stimulus sequence with a probability of 1∼4%. During the stimulus presentation, subjects were asked to search for the target images and ignore the non-target images in a subjective manner. To keep all original information, the dataset was the raw continuous data without any processing. On one hand, the dataset can be used as a benchmark dataset to compare the algorithms for target identification in RSVP-based BCIs. On the other hand, the dataset can be used to design new system diagrams and evaluate their BCI performance without collecting any new data through offline simulation. Furthermore, the dataset also provides high-quality data for characterizing and modeling event-related potentials (ERPs) and steady-state visual evoked potentials (SSVEPs) in RSVP-based BCIs. The dataset is freely available from http://bci.med.tsinghua.edu.cn/download.html.

## Introduction

Brain–computer interfaces (BCIs) provide a direct communication and control channel between the brain and external devices by analyzing neural activity, which has become one of the current study hot spots (Gao et al., 2014; Chen et al., 2015a; Han et al., 2020). Electroencephalogram (EEG) is the most widely used tool for BCIs because of its advantages such as non-invasiveness, low cost, and high temporal resolution (Stegman et al., 2020; Zhang et al., 2020). At present, remarkable progresses have been made in the performance and practicability of BCIs due to the optimization of the experimental paradigm, the improvement of the signal processing algorithm, and the application of the machine learning method (Chen et al., 2015b; Nakanishi et al., 2018; Zhang et al., 2018). Especially in recent years, the emergence of free open datasets has spared the time, money, and labor costs of data collection, thus providing convenience for the majority of BCI researchers and promoting the progress of algorithm development. The datasets covered many BCI paradigms such as steady-state visual evoked potentials (SSVEPs) (Wang et al., 2017; Lee et al., 2019), event-related P300 potentials (Abibullaev and Zollanvari, 2019; Vaineau et al., 2019), and motor imagery (Cho et al., 2017; Kaya et al., 2018). In addition, there are some open multimodal datasets for BCIs obtained synchronously with EEG (Lioi et al., 2019). As the broad audience of these datasets, researchers in a wide range of fields have contributed their intelligence to the BCI technology.

Rapid serial visual presentation (RSVP)-based BCI is a special type of BCI that detects target stimuli (e.g., letters or images) that are presented sequentially in a stream by detecting the brain’s response to the target. RSVP is the process of sequentially displaying images in the same spatial position at a high presentation rate with multiple images per second (such as 2–20 Hz) (Lees et al., 2017). In the applications that benefit from this paradigm, computers are unable to analyze and understand images with deep semantic and unstructured features as successfully as humans, and the manual analysis tools are slow, which makes the study of RSVP-BCI more and more popular in recent decades. RSVP-BCI has been used in counterintelligence, police, and health care that require professionals to review objects, scenes, people, and other relevant information contained in a large number of images (Huang et al., 2017; Singh and Jotheeswaran, 2018; Wu et al., 2018).

Different EEG components are associated with target and non-target stimuli (Bigdely-Shamlo et al., 2008; Cohen, 2014), and BCI signal processing algorithms have been used to recognize event-related potential (ERP) responses and link them to target images. The most commonly exploited ERP in RSVP-based BCI applications is the P300, ideally on a single-trial basis (Manor et al., 2016). In order to detect ERPs induced by target images, researchers have developed a variety of algorithms and evaluated them with the data collected independently (Sajda et al., 2003; Alpert et al., 2014; Zhao et al., 2019). Unfortunately, as far as we know, there is still a lack of a benchmark dataset for the RSVP-based BCI paradigm. It is always difficult to compare the performance of different algorithms with a small amount of data. One of the main difficulties in collecting a benchmark dataset is the large number of system parameters in RSVP-based BCIs (e.g., frequency of image presentation, target definition, target sparsity and identifiability, and number of trials and subjects). There is a great need to collect and publish a large benchmark dataset using the RSVP-based BCI paradigm.

This study provides an open dataset for BCI study based on the RSVP paradigm. The characteristics of this dataset are described as follows. (1) A large number of subjects (64 in total) were recorded. (2) A large number of stimulation image circles (16,000 for each subject) were included. (3) Complete data were provided with the original continuous data without any processing, including EEG data, electrode positions, and subjects information. (4) Stimulus events (onsets and offsets) were precisely synchronized to EEG data. (5) The 64-channel whole-brain EEG data were recorded. That means that this dataset contains a total of 64 subjects, 10,240 trials, 1,024,000 image circles, and 102,400 s of 64-channel EEG data. This dataset provides potential opportunities for developing signal processing and machine learning algorithms that rely on large amounts of EEG data. These features also make it possible to study the algorithms for ERP detection and the methods for stimulus coding with the dataset. In addition, through offline simulation, stimulus coding and target recognition methods can be jointly optimized toward the highest performance of an online BCI.

The rest of this paper is organized as follows. The *Methods* section introduces the experimental setup of data recording. The *Data Recording* section introduces the data records and other relevant information. The *Technical Validations* section introduces the basic methods in data analysis and gives three examples to illustrate how to use the dataset to study the methods of target detection in RSVP-based BCIs. The *Discussions and Conclusion* section summarizes and discusses the future work to improve the dataset.

## Materials and Methods

### Subjects

Sixty-four subjects (32 females; aged 19–27 years, mean age 22 years) with normal or corrected-to-normal vision were recruited for this study. Each subject signed a written informed consent before the experiment and received a monetary compensation for his or her participation. This study was approved by the Research Ethics Committee of Tsinghua University.

### Experimental Design

This study developed an offline RSVP-BCI system. A 23.6-inch liquid crystal display (LCD) screen was used to present visual stimuli. The resolution of the screen was 1,920 × 1,080 pixels, and the refresh rate was 60 Hz. The visual stimulus images were rendered within a 1,200 × 800-pixel square in the center of the screen. The screen area surrounding the stimuli image was gray colored [red green blue (RGB): (128, 128, 128)].

The stimulus program was developed under MATLAB (MathWorks, Inc.) using the Psychophysics Toolbox Ver. 3 (PTB-3) (Brainard, 1997). The stimulus images, downloaded from the Computer Science and Artificial Intelligence Library of MIT University, were street-view images of two categories: target images showing human and non-target images without human. During the experiment, subjects were asked to search for the target images and ignore the non-target images in a subjective manner. As previous studies have shown similar performance between motor and non-motor response tasks (Gerson et al., 2006), subjects in this study were required to make a manual button press to maintain attention once detecting target images in the RSVP task.

**FIGURE 1:**
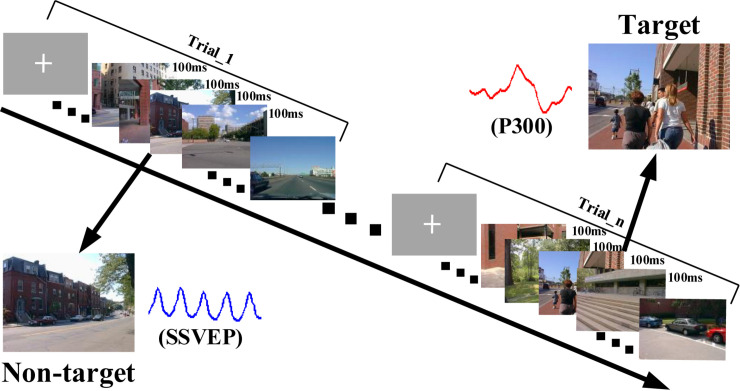
The time course of rapid serial visual presentation (RSVP) paradigm.

Figure 1 shows the time course of the RSVP paradigm. Each trial started with a blank for 0.5 s with a cross mark on the center of the screen, and subjects were asked to shift their gaze to the cross mark as soon as possible. The frequency of image presentation was set to 10 Hz (10 images per second).

**FIGURE 2:**
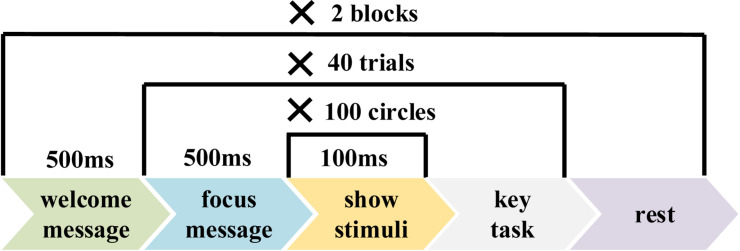
The parameter settings of the experiment for each group.

Figure 2 shows the parameter settings of the experiment for each group. Each group covered two blocks, each containing 40 trials. Each trial contained 100 images, including one, two, three, or four target images. Images in each trial were presented in a random order. At the beginning of each image’s presentation, a time marker named “event trigger” was sent by the stimulation program to mark the current stimulus image and was recorded on an event channel of the amplifier synchronized with EEG. There was a short key-controlled pause between trials. The duration of each block was about 10 min. There was an average rest time of about 5 min between two blocks to relieve subjects’ fatigue.

### Data Acquisition

Electroencephalogram data were recorded using the Synamps2 system (Neuroscan, Inc.) at a sampling rate of 1,000 Hz. All 64 electrodes were used to record EEG and were placed according to the international 10–20 system. The reference electrode, with the 10–20 electrode name of “Ref,” was located at the vertex. Electrode impedances were kept below 10 kΩ. During the experiment, subjects were seated in a comfortable chair in a dimly lit soundproof room at a distance of approximately 70 cm from the monitor. The EEG data were filtered from 0.15 to 200 Hz by the system. The power-line noise was removed by a notch filter at 50 Hz. It is worth to mention that the impedance of M1 and M2 electrodes (channels of 33 and 43) was higher than 10 kΩ for some subjects. We therefore suggest to select EEG data from the other 62 channels for analysis, and the EEG analysis in this study used the 62-channel data with the channel indices of [1:32 34:42 44:64] and removed the bad channels.

### Data Preprocessing

The dataset was the continuous data at a sampling rate of 250 Hz, and it was obtained from the raw EEG data (sampling rate at 1,000 Hz) after four times downsampling. For each of the datasets from 1 to 64 (sub1,…, sub64), EEG data contained four blocks, which were divided into two groups (namely, groups A and B) in chronological order. Each group contained two blocks, and each contained 40 trials. Each trial contained 100 circles, and each circle corresponded to one image. For each group, the two blocks were used for training and testing in the ERP-based target detection, respectively. In addition, a 10-fold cross-validation using both blocks 1 and 2 was performed to further evaluate the classification performance.

To verify the validity of the dataset, the continuous EEG data at a sample rate of 250 Hz were processed by a four-order Butterworth filter with a bandwidth of [2 30] Hz. EEG data epochs were extracted according to event triggers generated by the stimulus program. In this study, time 0 represented the beginning of each image stimulus period (marked by a trigger), and the EEG data corresponding to each image (namely, one circle) were intercepted within the time interval from −200 to 1,000 ms. The waveforms of ERPs and SSVEPs corresponding to target and non-target images were obtained using the averaged EEG data within the time interval of (−200 1,000) ms.

### Target Classification

Single-circle EEG data were firstly processed by spatial filtering methods, and then the target detection was realized by classification algorithms. Four spatial filtering methods, namely, common spatial pattern (CSP), SIgnal-to-noise ratio Maximizer (SIM), task-related component analysis (TRCA), and principal component analysis (PCA) whitening, were compared in this study. The effects of the number of components (from 1 to 50) of different spatial filtering methods on the classification performance were compared. The performance of spatial filtering was evaluated by the followed classification results of the classical Hierarchical Discriminant Component Analysis (HDCA) algorithm, which was adopted as a baseline measure of classification performance for single-circle EEG between target and non-target images (Gerson et al., 2006; Sajda et al., 2010). As a classical classification method widely used in RSVP-BCIs, HDCA algorithm realizes target images recognition based on spatial and temporal projection features of ERP signals. EEG data were firstly divided into 100-ms data segments, and then the feature extraction and classification were conducted according to the spatial and temporal characteristics of the data segments.

To evaluate the performance of the classification methods, four classification algorithms, namely, Support Vector Machine (SVM), Spatially Weighted Fisher linear discriminant (FLD)-PCA (SWFP), Discriminative Canonical Pattern Matching (DCPM), and HDCA, were compared based on this dataset. The EEG data used for single-circle classification were the data in the time interval of [0, t] ms, “t” might be 200, 300,…, 1,000 ms. SIM algorithm was used as a basic spatial filtering method before the performance comparison of the four classification algorithms.

### Performance Evaluation

**FIGURE 3:**
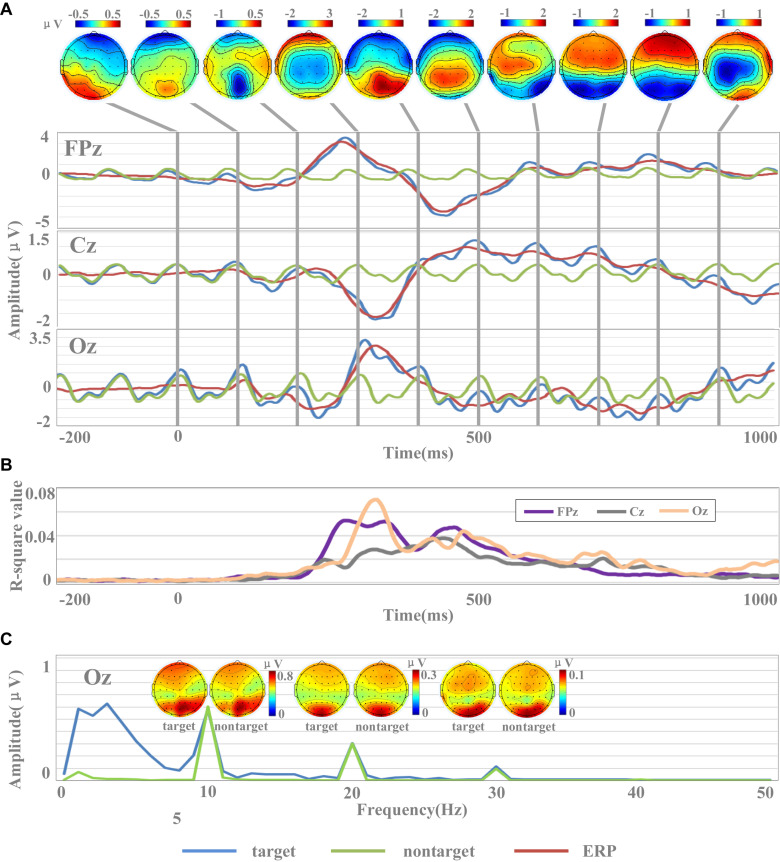
Waveforms and amplitude spectrum of EEG. **(A)** The temporal waveforms of EEG [including target images, non-target images, and event-related potential (ERP) data] and the scalp topographies of amplitudes of ERP. **(B)** The *r*-square values in channels of FPz, Cz, and Oz. **(C)** Spectral characteristics of EEG for target and non-target images.

*R*-square values for each time point were used to show the separability between target and non-target stimuli. For each subject, we selected all the target data and the same amount of non-target data randomly selected to calculate *r*-square values. For each time point, the input was composed of two one-dimensional vectors, which were composed of target data and non-target data, respectively. The *r*-square values of each subject were calculated, and the *r*-square values of all subjects were averaged to obtain the final results, as shown in Figure 3B.

Classification performance of single-circle EEG data for target and non-target circles was measured using the area under the receiver operating characteristic (ROC) curve (Fawcett, 2006). ROC curves are used when applications have an unbalanced class distribution, which is typically the case with RSVP-BCI, where the number of target stimulus is much smaller than that of non-target stimuli.

### Statistical Analysis

Statistical analyses were conducted using SPSS software (IBM SPSS Statistics, IBM Corporation). One-way repeated-measures analysis of variance (ANOVA) was used to test the difference in the classification performances among different algorithms. The Greenhouse–Geisser correction was applied if the data did not conform to the sphericity assumption by Mauchly’s test of sphericity. All pairwise comparisons were Bonferroni corrected. Statistical significance was defined as *p* < 0.05.

## Data Recording

### EEG Data

The dataset is freely available at http://bci.med.tsinghua.edu.cn/download.html. The dataset was the raw continuous data without any processing. It contains 128 MATLAB MAT files corresponding to data from all 64 subjects (approximately 15 GB in total). Data were stored as double-precision floating-point values in MATLAB. Each MAT file covers a group of EEG data. There are two sets of EEG data (groups A and B) for subjects from 1 to 64. The files were named as subject and group indices (i.e., sub1A.mat, sub1B.mat,…, sub64A.mat, sub64B.mat). For each file, the data loaded in MATLAB generate two 2-D matrices named “EEGdata1” (block1) and “EEGdata2” (block2) with dimensions of [64, L] (the two dimensions indicate “Electrode index,” “Time points,” respectively) and two 2-D matrices named “class_labels” and “trigger_positions” with dimensions of [2, 4000]. The parameter of L (the length of time points) might be different for different blocks. The two dimensions indicate “class labels,” in which “2” and “1” indicate “non-target images” and “target images,” respectively. Each circle corresponds to the EEG data of a visual stimulus image. For each group, the data matrix consists of 8,000 circles (100 circles × 40 trials × 2 blocks), and each circle consists of 64 channels of EEG data. A “Readme.txt” file explains the data structure and other task-related information.

### Electrode Position

The electrode positions were listed in a “64-channels.loc” file, which contained all channel locations in polar coordinates. Information for each electrode contained four columns: “Electrode Index,” “Degree,” “Radius,” and “Label.” For example, information on the first electrode was as follows: (“1,” “−18,” “0.51111,” and “FP1”), which indicated that the degree is −18, and the radius is 0.51111 for the first electrode (FP1). The electrode file can be used for topographic analysis by the topoplot() function in the EEGLAB toolbox (Delorme and Makeig, 2004).

## Technical Validations

### Temporal Waveform and Amplitude Spectrum Analysis

To evaluate the signal quality of the dataset, this study analyzed temporal waveform and amplitude spectrum of EEG across all subjects. EEG data were re-referenced to the average of all electrodes. Figure 3A shows the temporal waveform of averaged EEG across all subjects. Three representative midline electrodes (FPz, Cz, and Oz) were selected for temporal waveforms display. For each subject, all EEG data corresponding to target and non-target images were averaged. Then, the averaged target and non-target EEG data for each subject were averaged across all subjects. Finally, the cross-subject averaged EEG data corresponding to the non-target images were subtracted from that of the target images to generate the target-related ERP, as shown in Figure 3A. To better observe the temporal characters of the SSVEPs, the data were band-pass filtered between 2 and 30 Hz within the time window from −200 to 1,000 ms.

The EEG signals in this dataset were sensitive to target and non-target image stimuli, and the difference of the evoked EEG between the target and non-target image stimuli could be reflected by the ERP components within a short data length at specific brain regions. Figure 3A showed the temporal waveforms of EEG for target images, non-target images, and target-related ERP data. The waveform for non-target EEG is a near-sinusoidal signal at 10 Hz with the characteristics of SSVEP. The frequency and phase of the SSVEPs are stable over the 1.2-s stimulation time. The waveforms of ERP located at FPz and Oz showed obvious P300 (FPz: 3.18 μV, Oz: 2.54 μV) and N400 (FPz: −3.49 μV, Oz: −1.29 μV) components. Obviously, the latencies of P300 and N400 components in the prefrontal cortex were significantly smaller than those in the occipital cortex. For example, the latencies of the P300 component in FPz and Oz were 272 and 336 ms, while the latencies of the N400 component were 448 and 484 ms, respectively. While the ERP signal at Cz showed an obvious negative peak appeared around 300 ms (latency: 328 ms, amplitude: −1.29 μV). From the scalp topographies of amplitudes of ERP in Figure 3A, it could be found that the areas highly sensitive to ERP response were mainly located in the occipital region and the prefrontal region. For example, these two regions showed significant positive potentials at 300 ms and negative potentials at 400 and 500 ms. The sensitivity of ERPs for the electrode in the parietal region to the stimulation of target images was limited partly because the electrodes were close to the reference electrode.

The results of *r*-square values indicated the separability between target and non-target stimuli, as shown in Figure 3B. *R*-square values indicate the importance of features, and the larger the value, the greater the contribution to classification. In the time range of 0–200 ms, the *r*-square values of the three channels were close to 0, which indicated that the features did not contain information valid for classification. After the time of 200 ms, the *r*-square values of the three channels significantly increased, which was consistent with the emergence of the main components of ERP. For example, the *r*-square value of Oz reached the maximum value (0.07) at 340 ms, and at the same time, the ERP of Oz also reached the peak value (2.54 μV). Similar results were also found in Cz and Oz. These results indicated that the emergence of the main components of ERP was accompanied by a greater separability between target and non-target stimuli, and ERP was a potentially effective classification feature. Compared with Cz, the *r*-square values of FPz and Oz were larger, indicating that FPz and Oz contained more effective information and contributed more to classification.

The results of Figure 3 indicate that the rapid periodic stimulation in RSVP produces a brain response characterized by a “quasi-sinusoidal” waveform whose frequency components are constant in amplitude and phases. Figure 3C illustrates the amplitude spectra of EEG evoked by target and non-target images. EEG data were firstly averaged across all subjects, and then the spectrums were calculated by Fast Fourier Transform (FFT) method. As temporal waveforms in Figure 3A have shown the non-target EEG as a quasi-sinusoidal signal with stable frequency and phase, amplitude peaks of EEG at Oz can be observed at 10 Hz and its harmonic frequencies (i.e., 20, 30 Hz) from the frequency information in Figure 3C. The amplitudes of fundamental and harmonic components decreased sharply as the response frequency increased (fundamental: 0.60 μV, second harmonic: 0.30 μV, third harmonic: 0.10 μV). Since the signals were filtered from 2 to 30 Hz, the amplitudes in the frequencies above the fourth harmonic were closed to 0. Figure 3C also illustrates the scalp topographies of amplitude of target and non-target SSVEP at 10 Hz and its harmonic frequencies. Consistent with previous studies (Gao et al., 2014; Chen et al., 2015a), the occipital area shows the highest amplitude of SSVEPs. In addition to the occipital area, lower amplitude can also be observed at the prefrontal area for components related to stimulus frequency (at 10 and 20 Hz). These characters show very robust and reliable frequency features for the fundamental and harmonic SSVEP components in the dataset and suggest that the RSVP stimulation at 10 Hz in this dataset was stable and reliable.

As the phase characteristic of SSVEPs is synchronous (Figure 3A) and the amplitude characteristic is approximate (Figure 3C) between target and non-target EEG, target-related ERP signal can be extracted by subtracting non-target EEG from target EEG signals. There were obvious similarities and differences between EEG signals evoked by target images and non-target images in frequency domain. The EEG signals of target images have similar amplitudes of EEG components at the fundamental and harmonic frequencies (fundamental: 0.58 μV, second harmonic: 0.31 μV, third harmonic: 0.11 μV) with that of non-target images. Furthermore, the EEG of the target images contained more powerful low-frequency components (<10 Hz), which were related to ERP. This character suggests that the spectral characteristics provide useful information for the detection of target images.

### Evaluating the Performance of Spatial Filtering Methods

**FIGURE 4:**
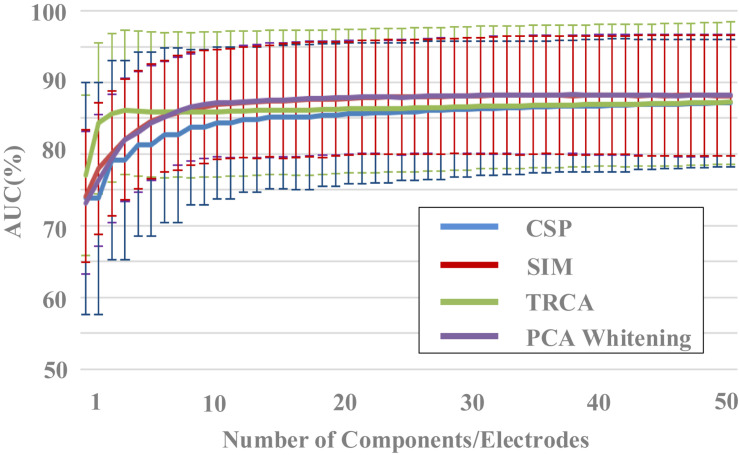
The effect of components number on classification performance (block 1 for training, block 2 for testing).

Spatial filtering aims to remove signal noise and extract task-related brain activities by using the spatial correlation information of EEG and is frequently applied as a preprocessing method. It has been widely used in EEG-based BCIs. Figure 4 indicated the performance of different spatial filtering methods in the target/non-target classification task based on the HDCA classification algorithm. Four filtering methods were used to enhance classification performance: CSP, SIM, TRCA, and PCA whitening. CSP consists of finding an optimum spatial filter to maximize the variance difference between two groups of EEG, so as to obtain effective feature vectors for classification (Lotte and Guan, 2011). The algorithm of SIM can be intuitively interpreted as maximizing the signal-to-noise ratio (SNR) in the source space and is an effective tool for spatiotemporal analysis of ERPs (Wu and Gao, 2011). TRCA is the method that extracts task-related components efficiently by maximizing the reproducibility during the task period and can be applied to enhance SNRs of time-locked EEG components such as ERPs (Nakanishi et al., 2018). PCA whitening is a simple and standard procedure to reduce dimension of the data, and it can reduce the complexity by reducing the number of parameters to be estimated (Hyvarinen and Oja, 2000).

After the spatial filtering processes, we adopted the HDCA method, which has been widely used in EEG target image detection based on RSVP paradigm, to classify the target and non-target images. For the two blocks in each group of the dataset, EEG data in block 1 were used for training (i.e., to determine parameters of the algorithms), and EEG data in block 2 were used for testing. In addition, both block 1 and block 2 were used for a 10-fold cross-validation to further evaluate the classification performance. Data from all the 62 electrodes were used as the input to the feature extraction and classification analysis. EEG data were firstly divided into 100-ms data segments. Then the feature extraction and classification were conducted according to the spatial and temporal characteristics of the data segments.

The effect of components number of the four spatial filtering methods on classification performance was evaluated. The data length was 400 ms [time window (0 400) ms]. By setting the number of components in the spatial filtering methods (from 1 to 50), the variation of classification performance with the number of components can be obtained (Figure 4). The classification performance increased as the number of components increased, especially when the components number was less than 10. For example, the area under the curve (AUC) results for the SIM method were 74.1% ± 9.2%, 78.0% ± 9.2%, 80.0% ± 8.7%, 82.0% ± 8.4%, 83.4% ± 8.3%, 84.6% ± 8.0%, 85.3% ± 7.8%, 85.7% ± 8.0%, 86.3% ± 7.9%, 86.6% ± 7.9% for the components number from 1 to 10, respectively. Especially in the case a small number of components, the TRCA algorithm had the best classification performance. For example, the AUC of TRCA was 77.0, 84.3, and 85.6 as the components number from 1 to 3, respectively, which is far larger than other methods. When the number of components is more than 10, the classification performance no longer changes significantly for all the four methods, and the methods of SIM and PCA whitening show the best performance (SIM: 87.9%, PCA whitening: 88.0%).

A one-way repeated-measures ANOVA showed that there was a statistically significant difference in accuracies among the four spatial filtering methods for the component numbers of 1 [F(2.110,268.008) = 4.648, *p* = 0.009] and from 2 to 50 (*p* < 0.001). Pairwise comparisons showed that the classification accuracies of TRCA were significantly higher (*p* < 0.05) than that of CSP for the component numbers from 2 to 50 and were significantly higher than that of SIM and PCA whitening for the component numbers from 1 to 6. The classification accuracies of SIM and PCA whitening were significantly higher (*p* < 0.05) than that of CSP for the component numbers from 6 to 50 and were significantly higher than that of TRCA for the component numbers from 11 to 50.

**FIGURE 5:**
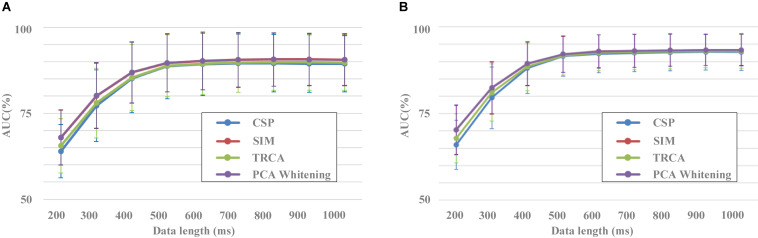
Performance of different data lengths for spatial filtering methods **(A)** Block 1 for training and block 2 for testing. **(B)** Result of 10-fold cross-validation using both blocks 1 and 2.

Figure 5 shows the results of classification performance for the four spatial filtering methods with different data lengths of EEG. The number of components for the four spatial filtering methods was set to 30. Two validation methods were used, that is, block 1 for training and block 2 for testing (Figure 5A) and a 10-fold cross-validation using both blocks 1 and 2 (Figure 5B). For each spatial filtering method, the classification accuracy increased obviously as the data length increased when it was less than 500 ms. For example, in Figure 5A, the average results of SIM for all subjects were 67.7% ± 7.3%, 80.5% ± 8.8%, 88.1% ± 8.2%, and 91.1% ± 7.2% with the data length from 200 to 500 ms, respectively. The changes of accuracy results were no longer significant when the length of EEG data increased to 600 ms and above.

In addition, there was a significant difference in the classification performance among the different spatial filtering methods. The CSP method corresponded to the worst classification performance, followed by the TRCA method. SIM and PCA whitening methods had higher classification performance with no statistically significant difference. For example, in Figure 5A, the classification results were 77.2% ± 10.1%, 78.3% ± 9.4%, 80.5% ± 8.8%, and 80.7% ± 8.8% for the data length of 300 ms in the conditions of CSP, TRCA, SIM, and PCA whitening, respectively. The statistical difference among CSP, SIM, and TRCA was no longer significant when the data length was more than 500 ms. Meanwhile, the high classification results based on EEG with short data lengths indicated that the dataset was collected in a well-designed experimental environment, and the collected EEG data were of high quality.

The 10-fold cross-validation method showed similar results to the original verification method by blocks, i.e., SIM and PCA whitening performed best among the four spatial filtering methods, and HDCA was the best among the four classification methods. The difference between the two validation methods was that the accuracies and variances of the 10-fold cross-validation method were slightly higher and smaller than the method by blocks, respectively. For example, the classification results for CSP, SIM, TRCA, and PCA whitening were 66.0% ± 7.0%, 70.2% ± 7.1%, 67.9% ± 7.0%, and 70.3% ± 7.1% and 63.4% ± 7.1%, 67.7% ± 7.3%, 65.4% ± 7.3%, and 67.8% ± 7.3% for 10-fold cross-validation method and validation method by blocks, respectively. This was due to the fact that the 10-fold cross-validation method used more data for training than the original verification method by blocks. Since the two validation methods have shown similar results, we only chose the classification results of the validation method by blocks to perform the statistical analysis in this study.

A one-way repeated-measures ANOVA showed that there was a statistically significant difference in accuracies among the four spatial filtering methods for the data length of 200 ms [F(1.326,168.403) = 76.929, *p* < 0.001], 300 ms [F(1.324,168.179) = 115.527, *p* < 0.001], 400 ms [F(1.204,152.967) = 128.453, *p* < 0.001], 500 ms [F(1.333,169.256) = 124.089, *p* < 0.001], 600 ms [F(1.247, 58.402) = 131.426, *p* < 0.001], 700 ms [F(1.248,158.528) = 101.262, *p* < 0.001], 800 ms [F(1.409,178.955) = 100.214, *p* < 0.001], 900 ms [F(1.404,178.285) = 99.643, *p* < 0.001], and 1,000 ms [F(1.350,171.387) = 102.250, *p* < 0.001]. Pairwise comparisons showed that the classification accuracies of SIM and PCA whitening were significantly higher (*p* < 0.001) than those of CSP and TRCA for the data length from 200 to 1,000 ms. The classification accuracies of TRCA were significantly higher (*p* < 0.01) than that of CSP for the data length from 200 to 300 ms and were significantly lower (*p* < 0.001) than that of CSP for the data length from 400 to 1,000 ms. There was no significant difference between SIM and PCA whitening for the performance of classification.

### Evaluating the Performance of Classification Methods

**FIGURE 6:**
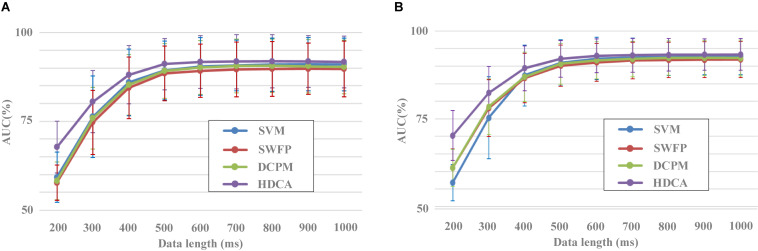
Performance of different classification methods with different data lengths. **(A)** Block 1 for training and block 2 for testing. **(B)** Result of 10-fold cross-validation using both blocks 1 and 2.

In addition to the evaluation of spatial filtering methods, the dataset can also be used to evaluate the performance of classification methods. Figure 6 indicated the performance of different classification methods with the EEG data length from 200 to 1,000 ms. After preprocessing with the SIM method, EEG data for each image were classified by four different algorithms including SVM, SWFP, DCPM, and HDCA. SVM finds a separating hyper-plane that maximizes the margin between the two classes. SWFP is based on a two-step linear classification of event-related responses using FLD classifier and PCA for dimensionality reduction (Alpert et al., 2014). DCPM performs well in classifying the miniature AVePs by first suppressing the common-mode noise of the background EEG and then recognizing canonical patterns of ERPs (Xiao et al., 2020). Two validation methods were used, that is, block 1 for training and block 2 for testing (Figure 6A), and a 10-fold cross-validation using both blocks 1 and 2 (Figure 6B). As shown in Figure 6A, HDCA had the best classification performance, while the other three algorithms had approximately a similar classification performance. This was especially true when the data length was less than 500 ms. For example, the AUC results for HDCA were 67.7% ± 7.3%, 80.5% ± 8.8%, 88.1% ± 8.2%, and 91.1% ± 7.2% for single-circle EEG classification between target and non-target images with the data length of 200, 300, 400, and 500 ms, respectively. When the data length is greater than 500 ms, the performance of the four classification algorithms is similar, while the classification performance of the HDCA algorithm is still the best. Figure 6B indicated the similar results as Figure 6A, and the only difference was that the SVM method performed the worst.

A one-way repeated-measures ANOVA based on the validation method by blocks showed that there was a statistically significant difference in accuracies among the four classification methods for the data length of 200 ms [F(2.124,269.799) = 144.651, *p* < 0.001], 300 ms [F(1.942,246.670) = 55.645, *p* < 0.001], 400 ms [F(2.095,266.046) = 42.243, *p* < 0.001], 500 ms [F(2.183,277.251) = 38.436, *p* < 0.001], 600 ms [F(2.362,299.935) = 35.408, *p* < 0.001], 700 ms [F(3,381) = 27.146, *p* < 0.001], 800 ms [F(2.820,358.107) = 33.019, *p* < 0.001], 900 ms [F(2.601,330.287) = 29.985, *p* < 0.001], and 1,000 ms [F(3,381) = 32.344, *p* < 0.001]. Pairwise comparisons showed that the classification accuracies of HDCA were significantly higher (*p* < 0.001) than that of SVM, SWFP, and DCPM for the data length from 200 to 1,000 ms. The classification accuracies of SVM were significantly higher (*p* < 0.05) than that of SWFP for the data length from 400 to 1,000 ms and were significantly higher (*p* < 0.05) than that of DCPM for the data length from 900 to 1,000 ms. The classification accuracies of DCPM were significantly higher (*p* < 0.01) than that of SWFP for the data length from 300 to 1,000 ms.

### Evaluating the Performance of Cross-Subject Zero-Training Methods

The dataset can be used to study zero-training classification methods of RSVP-based BCIs. To improve the performance of the system, most of the current RSVP-based BCIs adopt supervised feature extraction and classification algorithms that require system calibration. The long time in training data collection and algorithm template extraction processes bring challenges to system practicability and user experience. With benefits from the large scale of the dataset that contains a total of 64 subjects, 10,240 trials, 1,024,000 image circles, and 102,400 s of 64-channel EEG data, it is possible to extract common information of EEG for target classification. A cross-subject strategy can be used to design zero-training algorithms suitable for target identification in the RSVP paradigm.

**FIGURE 7:**
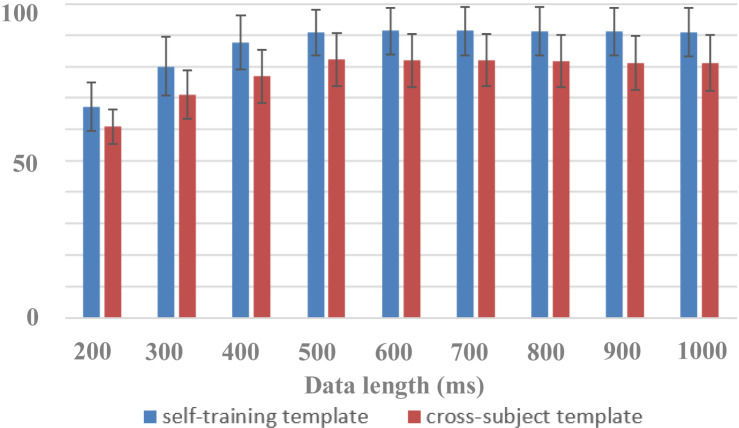
Performance of cross-subject zero-training and self-training methods (block 1 for training and block 2 for testing).

In this paper, the dataset was used to design a zero-training classification algorithm based on a cross-subject template. The performance was estimated using a leave-one-subject-out cross-validation. EEG data of each subject were trained separately to obtain his or her algorithm template parameters for the HDCA algorithm. In the testing session, by using cross-subject template, the EEG classification performance of one subject was determined by the voting results of the algorithm templates of the other 63 subjects, all of whom had an equal voting weight. Figure 7 showed the performance of cross-subject zero-training method using the HDCA algorithm. Pairwise comparisons showed that the classification accuracies of the traditional self-training method were significantly higher than that of the cross-subject method for the data length of 200 ms [F(1,127) = 83.0101, *p* < 0.001], 300 ms [F(1,127) = 164.440, *p* < 0.001], 400 ms [F(1,127) = 195.524, *p* < 0.001], 500 ms [F(1,127) = 137.263, *p* < 0.001], 600 ms [F(1,127) = 143.973, *p* < 0.001], 700 ms [F(1,127) = 139.003, *p* < 0.001], 800 ms [F(1,127) = 139.555, *p* < 0.001], 900 ms [F(1,127) = 151.889, *p* < 0.001], and 1,000 ms [F(1,127) = 141.892, *p* < 0.001]. Although the performance of cross-subject method was lower than the traditional self-training method, it still achieved good performance for more than 80% of AUC when the data length was more than 400 ms. For example, the AUCs were 82.2% ± 8.4% and 90.8% ± 7.4% by using cross-subject and self-training templates, respectively, when the data length was 500 ms. The results indicated that a variety of cross-subject information could be mined from the dataset. By using the dataset appropriately, we can effectively design algorithms that do not require system calibration. With the mining of more effective information contained in the dataset, it is believed that the performance of zero-training algorithm can be further improved and even closer to the performance of the training methods. This dataset provides sufficient data for the development of zero-training algorithms that can promote the practical application of RSVP-based BCIs.

## Discussion and Conclusion

This study presents a benchmark dataset for studying RSVP-based BCIs. Distinct ERP and SSVEP features in temporal, frequency, and spatial domains prove the high quality of data. The examples on evaluating classification performance further demonstrate high efficiency of the dataset for evaluating methods in target image detection.

In this study, continuous image stimulation was divided into periodic segments to resist fatigue and ensure the high quality and reliability of EEG signals. To reduce the interference of blinking on EEG, subjects were instructed to blink between trials rather than within the image sequence of stimuli, and they initiated the next trial by pressing a button. At the same time, subjects were given enough rest between blocks until they felt comfortable to start the next block. In this study, no strict experimental interruption time was set, which fully guaranteed the quality of EEG signals. The impact of rest time can be considered in future practical applications.

Besides the above technical validations proposed in this study, the dataset can be further analyzed in a variety of different ways. In fact, although remarkable progresses have been made in RSVP-BCI, there are still many defects to be solved. Firstly, the parameters of RSVP-BCI need to be optimized to meet different application requirements; secondly, the characteristics of SSVEP and ERP that are evoked by the RSVP paradigm require further investigation; thirdly, the separation methods of SSVEP and ERP are not effective. This dataset can be used for developing methods to address these limitations. On one hand, the dataset can be used to design system diagrams toward different applications. The optimization of parameters is very important for the design and implementation of a practical BCI system (Zhang and Gao, 2019; Lees et al., 2020). For example, the effect of time interval between target images on EEG characteristics can not only inspire the design of optimal RSVP stimulation paradigm but also deepen the understanding of attentional blink. Regarding the phase of the EEG, although other experimental paradigms such as SSVEP-BCIs have already shown indicators of phase character of evoked EEG such as latency, very few studies based on RSVP-BCIs explored phase characters. The evoked EEG phase in the RSVP paradigm must contain higher cognitive mechanisms, which makes the relevant research more significant. Besides, the number of electrodes and electrode locations can be optimized using the 64-channel dataset. On the other hand, the dataset can be used to develop computational models for ERPs and SSVEPs. The high SNR of ERP and SSVEPs from the dataset could be helpful for exploring the intrinsic properties of ERP and SSVEP harmonics. For example, the way to characterize the phases of the fundamental and harmonic SSVEP components still remains unknown. Furthermore, it is of great scientific significance to study the methods for separating ERP and SSVEP signals and the temporal dynamics and phase relations between them. The problem has not been well solved so far, and this dataset provides rich resources for the related studies.

In future work, the dataset can be improved in the following directions. First, data evoked by stimulus images with different frequencies will be included. In this study, the stimulation frequency was set to the most commonly used 10 Hz. EEG data with different frequencies may help to reveal the effect of workload on EEG. Secondly, more types of target sparsity will be included. As the target sparsity is set as 1∼4% in this study, the probability of target images can be further increased to verify the relationship between target density and the EEG signals. Third, data records from the same group of subjects on different days will be provided for developing the session-to-session transfer approach (Zhao et al., 2019), which can facilitate system calibration in an online BCI.

## Data Availability Statement

The datasets presented in this study can be found at: http://bci.med.tsinghua.edu.cn/download.html.

## Ethics Statement

The studies involving human participants were reviewed and approved by The Research Ethics Committee of Tsinghua University. The patients/participants provided their written informed consent to participate in this study.

## Author Contributions

YW, LZ, and XG designed the research. SZ performed the research. SZ and YW analyzed the data. All authors wrote the manuscript and contributed to the article and approved the submitted version.

## Conflict of Interest

The authors declare that the research was conducted in the absence of any commercial or financial relationships that could be construed as a potential conflict of interest.
